# Clinical and genetic characterization of a Danish family with North Carolina macular dystrophy

**Published:** 2010-12-09

**Authors:** Thomas Rosenberg, Ben Roos, Thorkild Johnsen, Niels Bech, Todd E. Scheetz, Michael Larsen, Edwin M. Stone, John H. Fingert

**Affiliations:** 1 National Eye Clinic, Kennedy Center, Glostrup, Denmark; 2 Gordon Norrie Center of Genetic Eye Diseases, Glostrup, Denmark; 3 Department of Ophthalmology and Visual Sciences, University of Iowa Carver College of Medicine, University of Iowa, Iowa City, IA; 4 Department of Ophthalmology, Glostrup Hospital, University of Copenhagen, Glostrup, Denmark; 5 Howard Hughes Medical Institute, Iowa City, IA

## Abstract

**Purpose:**

To describe the phenotype of a family with an autosomal dominant macular dystrophy and identify the chromosomal location of the gene that causes this phenotype.

**Methods:**

Twelve members of a three-generation family underwent routine clinical examination, including fundus photography. Four of the patients underwent extended examination with Goldmann perimetry, full-field electroretinogram, dark adaptation, and color vision testing, and two patients underwent optical coherence tomography and fundus autofluorescence examination. DNA samples were obtained from 12 family members and 3 spouses and genotyped at the known North Carolina Macular Dystrophy (NCMD) locus on chromosome 6q (MCDR1: OMIM 136550) using short tandem repeat polymorphisms. DNA samples were subsequently examined with a genome-wide scan of single nucleotide polymorphisms and the genotypes that were produced were studied with linkage and haplotype analyses.

**Results:**

The 10 affected family members had clinical findings of macular lesions that are typical for NCMD. The small drusen-like yellowish lesions of mild NCMD were hyperautofluorescent. Hyperpigmented foveal lesions were surrounded by a zone of confluent hyperautofluorescence. Linkage analysis of short tandem repeat polymorphism genetic markers excluded the NCMD locus on chromosome 6. However, analysis of single nucleotide polymorphism genotypes from a genome-wide scan showed that NCMD in our pedigree is linked to a region on chromosome 5p that overlaps the previously mapped macular dystrophy (MCDR3) locus with a maximum log of the odds (LOD) score of 2.69 at a recombination fraction of 0.00 (markers D5S406, D5S1987, and D5S2505).

**Discussion:**

We report the first pedigree with NCMD from Scandinavia, and the first confirmation that a gene for this condition is located on chromosome 5p13-p15. The bright elements or lesions typical of NCMD differed from drusen in that no sign of accumulation of material between the retinal pigment epithelium and Bruch’s membrane was seen. While the present study has found indications that the elements are located in the outermost layers of the retina, their precise location remains to be identified directly.

## Introduction

North Carolina macular dystrophy (NCMD) is an autosomal dominant dystrophy that was first reported as hereditary macular degeneration and aminoaciduria by Lefler, Wadworth, and Sidbury [1]. Extensive genealogical investigations in this and other families from the United States established a common ancestry to two Irish brothers who settled in North Carolina in the 1830s [2], although at the time the macular dystrophy was termed dominant foveal dystrophy [3] or central areolar pigment epithelial dystrophy [4]. The original North Carolina pedigree now consists of over 5,000 individuals (see review by Small [5]). Subsequently, families with a similar spectrum of phenotypes have been reported from Europe (the UK, Germany, and France) [6-8], Belize [9], and Korea [10].

The main characteristics of the disease consist of: symmetric, tiny drusen-like, yellowish deposits confined to the foveal region with little or no impact on visual acuity (Grade 1) [11]; confluent yellow flecks with intermediate visual impairment (Grade 2); or colobomatous macular chorioretinal atrophy often surrounded by pigment aggregations and associated with moderate to severe visual impairment (Grade 3). NCMD is generally nonprogressive and the grading does not reflect successive stages of progression [6]. Visual acuity is often astonishingly preserved compared with the severe appearance of the retinal lesion. Some individuals, however, develop subretinal neovascular membranes, resulting in fibrosis and late vision loss [12]. Peripheral drusen formation in a linear configuration without functional significance has also been frequently reported [5-7,11]. Peripheral visual fields, full-field electroretinogram (ERG), and electrooculogram (EOG) are rarely affected, while multifocal ERG demonstrates the reduction of central cone responses. Additional functional studies have further characterized the ocular phenotype of NCMD [6,13,14].

Linkage studies of NCMD pedigrees mapped a disease-causing gene to a locus (MCDR1, OMIM 136550) on chromosome 6q16 [15,16], which was later narrowed to ~3 cM (1.8 mb) [5,17]. Despite comprehensive sequencing of all 11 known genes within this interval, the gene involved has not yet been identified [18]. In the majority of pedigrees, NCMD is linked to the inheritance of a disease-causing gene in the chromosome 6q MCDR1 locus, and a common ancestral haplotype has been identified in most American families [5]. However, not all NCMD families are linked to the MCDR1 locus on chromosome 6q. Linkage analysis of a clinically well documented family with a phenotype indistinguishable from MCDR1 mapped another disease-causing gene to a chromosome 5p15 locus (MCDR3, OMIM 608850) [19]. Furthermore, the gene that causes NCMD and progressive sensorineural hearing loss in another large pedigree has been mapped to chromosome 14q [20]. Lastly, an additional family with a phenotype similar to NCMD has been linked to chromosome 6q14 adjacent to, but distinct from the MCDR1 locus, providing further evidence for genetic heterogeneity [21]. Here, we report the clinical characterization and linkage analysis of a Danish pedigree with features of NCMD.

## Methods

The study adhered to the Declaration of Helsinki and was approved by the local ethics committee. All patients gave their written consent after being informed about the purpose and implications of the investigation.

Two patients from the same family were independently referred to the National Eye Clinic for the Visually Impaired (NEC) for diagnostic examination. The findings initiated a supplementary examination of additional family members. All patients underwent a standard examination, including a review of their visual symptoms; assessment of refraction, Snellen visual acuity, ocular alignment and motility; slit lamp examination; ophthalmoscopy; and color fundus photography of macular lesions. Some patients underwent supplementary examination with Goldmann perimetry (object size IV4e and I4e); color vision screening (Ishihara’s 38 pseudoisochromatic plates and Lanthony tritan plates); and full-field ERG (ffERG; Nicolet Biomedical Instruments, Madison, WI), according to the recommendations by the International Society for Clinical Electrophysiology and Vision [22]. In addition, two of the patients underwent fundus autofluorescence imaging using a confocal scanning laser ophthalmoscope and optical coherence tomography instrument (Spectralis; Heidelberg Engineering, Heidelberg, Germany). Blood samples were obtained from family members and DNA was prepared from the blood using standard methods.

Pedigree members were first genotyped with short tandem repeat polymorphism (STRP) genetic markers flanking the MCDR1 locus. Genotyping with STRP genetic markers was conducted using standard methods, as previously described [23]. Twelve and one half nanograms of each patient's DNA was used as template in an 8.35 μl polymerase chain reaction (PCR) containing 1.25 μ of X10 buffer (100-mMol/l TRIS-hydrochloride [pH, 8.8]; 500-mMol/l potassium chloride; and 15-mMol/l magnesium chloride, 0.01% wt/vol gelatin); 200 μl each of deoxycytidine triphosphate, deoxyATP, deoxyguanosine triphosphate and deoxythymidine triphosphate; 1 pmol of each primer; and 0.25-U Taq polymerase (Perkin-Elmer Cetus, Norwalk, Conn). Samples were incubated in a DNA thermocycler for 35 cycles under the following conditions: 94 °C for 30 s, 55 °C for 30 s, and 72 °C for 30 s. After amplification, 5 μ of stop solution (95% formamide, 10-mMol/l sodium hydroxide, 0.05% bromophenol blue, and 0.05% xylene cyanol) was added to each sample. Amplification products were then denatured and electrophoresed on 6% polyacrylamide gels at 60W for approximately 3 h. Following electrophoresis, gels were silver stained as previously described [24] and genotypes were scored by inspection. Next, a genome-wide scan was performed with Affymetrix microarrays (*Sty1* array of the GeneChip Human Mapping 500K Array Set; Affymetrix, Santa Clara, CA), which interrogate 238,304 single nucleotide polymorphisms (SNPs). Sample processing and labeling were performed using the manufacturer’s instructions. The arrays were hybridized, washed, and scanned in the University of Iowa DNA core facility. Array images were processed with GeneChip DNA Analysis software (Affymetrix). Microarray data were analyzed and multipoint nonparametric linkage scores were calculated using the EasyLinkage software package [25]. Pairwise linkage analysis using STRP markers was performed with the MLINK and LODSCORE programs, as implemented in the FASTLINK (v2.3) version of the LINKAGE software package [26-28]. Penetrance and disease gene frequency were set to 99% and 0.01% respectively. For each STRP marker, the allele frequencies were assumed to be equal. True allele frequencies could not be reliably estimated from the small number of spouses in the pedigree. To show that the assumption of the equal allele frequencies would not significantly affect our linkage results, we recalculated the log of the odds (LOD) scores using allele frequencies for the “affected” allele of the most tightly linked marker (D5S1987) ranging from 0.01 to 0.5. The Z_max_ for D51987 was 2.69 when the “affected” allele frequency was arbitrarily set to 50%, suggesting that linkage is present despite assumptions about allele frequencies.

## Results

### Clinical examinations

**Figure 1:**
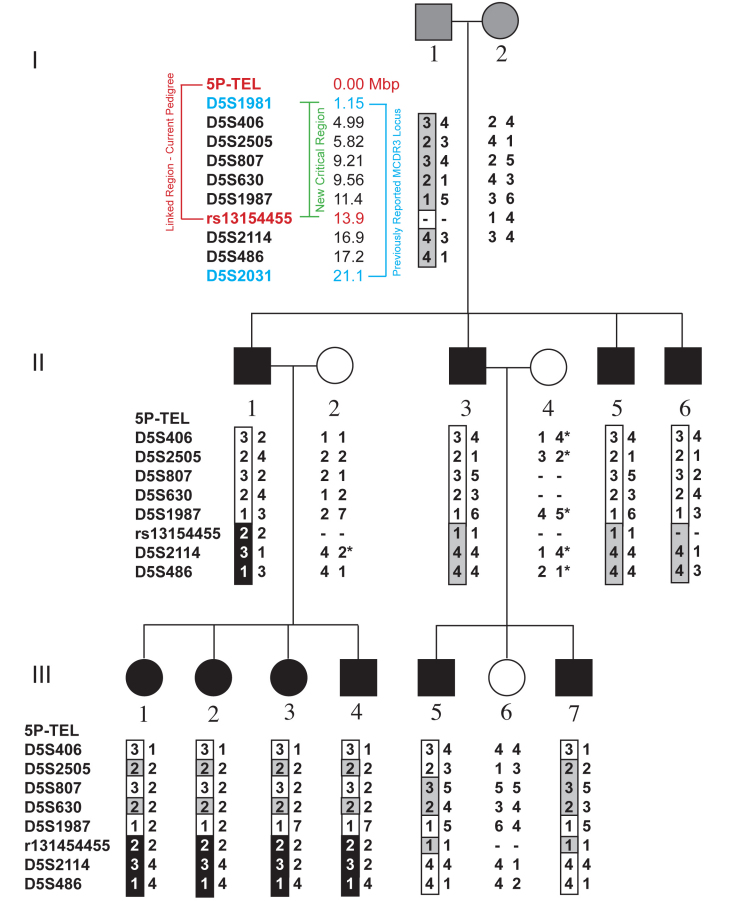
Pedigree of a Danish family with a North Carolina macular dystrophy-like phenotype. Individuals found to be clinically affected with North Carolina macular dystrophy (NCMD) are represented by black symbols, while unaffected individuals are depicted with open symbols. Family members with unknown affection status and family members that did not meet full criteria for a clinical diagnosis of NCMD are indicated with gray symbols. Individuals that are deceased are marked with a slash. Genotypes from seven genetic markers on the long arm of chromosome 5 are shown with the most telomeric markers at the top. The genotypes detected for each marker are shown beneath each subject’s pedigree symbol to the right of the marker name. The linked allele of each marker is on the left and boxed. If analysis of the genotypes revealed a meiotic recombination, the box around the recombinant allele is shaded black; if no recombination was detected, the box has been left unshaded. If the presence of a recombination could not be determined, the box is shaded gray. Genotypes that could not be determined are indicated with a dash, while those genotypes that were inferred are indicated with an asterisk. The positions of the markers on chromosome 5 are shown based on the GRCh37/hg19 build of the human genome.

**Figure 2:**
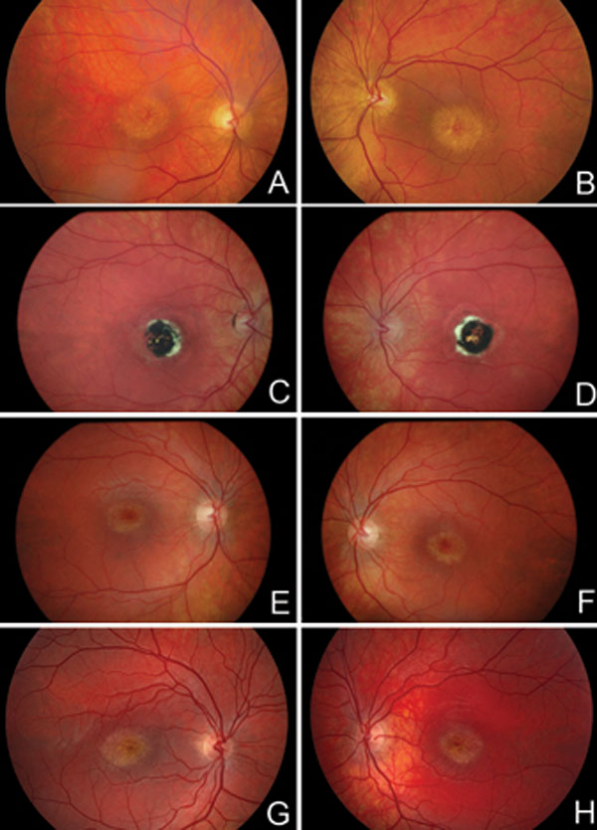
Selected color fundus pictures of the North Carolina macular dystrophy pedigree. Right (left column) and left (right column) macular regions are shown for four affected individuals: **A**-**B**, patient II-1; **C**-**D**, patient II-6; **E**-**F**, patient III-2; **G**-**H**, patient III-3. The macular lesions **A**-**B** and **E**-**H** are grade 1, while the lesions in **C**-**D** are grade 3.

Members of a three-generation pedigree (Figure 1) received complete eye examinations and 10 members were found to be clinically affected with NCMD. Affected members of the family exhibited typical features of NCMD, including characteristic macular lesions (Figure 2), relatively good visual acuity, and normal visual fields. The clinical findings are summarized in Table 1.

**Table 1:** Clinical findings in examined family members.

**Subject**	**Diagnosis**	**Age at exam**	**Snellen VA R/L**	**Refract. R/L**	**Color vision**	**Visual fields**	**ERG**	**Additional findings**
II-1	NCMD	48	1.0 0.2	+0.75 +2.00	N.D.	Normal	Normal	Amblyopia L.E.
II-2*	NCMD	46	0.8 0.8	+2.50–1,5x35° +2.00	N.D.	N.D.	N.D.	Hemorrhages and cotton wool spots due to undiagnosed hypertension
II:3*	NCMD	44	1.0 1.0	+2.25–0.5x110° +2.00–0.5x75°	N.D.	N.D.	N.D.	Exotropia
II-4	NCMD	33	0.5 0.4	−1.00x90° −1.00–1.00x80°	Ishihara 2 errors, Lanthony tritan normal	Normal	N.D.	Non-visual reading difficulties
III-1	NCMD	22	1.0 1.0	−0.50 −0.50	N.D.	N.D.	N.D.	N.D.
III-2	NCMD	20	1.0 1.0	Emmetropia Emmetropia	Ishihara, Farnsworth D15, Lanthony tritan: normal	N.D.	N.D.	N.D.
III-3	NCMD	12	0.66 1.33	+3.00–0.25x110° +1.25–0.25x25°	Ishihara, Lanthony tritan: normal	Slightly constricted lower right quadrant	Normal	Slight photophobia
III-4	NCMD	10	1.33 1.33	Emmetropia Emmetropia	N.D.	N.D.	N.D.	N.D.
III-5*	NCMD	24	1.0 1.0	Emmetropia Emmetropia	N.D.	N.D.	N.D.	N.D.
III-6*	Normal	21	1.0 1.0	+4.00–1.00x100° +4.00–1.25x75°	N.D.	N.D.	N.D.	N.D.
III-7*	NCMD	17	1.0 0.8	+4.50–2.50x25° +4.45–2.50x160°	N.D.	N.D.	N.D.	Left esotropia

Subject numbers refer to the pedigree Figure 1. Individuals marked with an asterisk were examined in private practice, while those without asterisks were examined at the National Eye Clinic. The presence or absence of macular lesions typical for NCMD was based on inspection of fundus pictures. V.A. Snellen visual acuity, R/L right/left, N.D. no data.

### Case reports

The proband, a 13-year-old school girl (Figure 1, III-3), was referred to NEC for diagnostic examinations due to a macular disorder. Her only visual complaint was moderate photophobia. There was no known family history of eye disease. The examination showed a best corrected Snellen visual acuity of 0.66 OD and 1.33 OS, hyperopia, and normal color discrimination. Kinetic visual fields (Goldmann perimetry with I4e and IV4e targets) were slightly constricted and automated perimetry (Octopus 101) showed bilateral defects in the lower right quadrant. Ophthalmoscopy revealed slightly oval-shaped bilateral macular lesions consisting of fine, densely packed, grainy depigmentations resembling small laminar drusen (Figure 2G,H). Scotopic and photopic ffERG showed normal waveforms with normal amplitudes and implicit times. At a follow-up examination two years later, the findings were unchanged. At this time, examination of the proband’s two elder sisters (III-1 and III-2), and a younger brother (III-4) who did not have any visual complaints, revealed the same macular lesions.

The proband’s paternal uncle (Figure 1, II-6) had a history of subnormal vision that was detected at seven years of age with screening tests at school, at which time “toxoplasmosis-like” macular scars were noted in both eyes. At 33 years of age, he was examined at NEC. He had no visual complaints and a best corrected Snellen visual acuity of 0.5 OD and 0.4 OS. However, with questioning, he reported difficulties with words disappearing during reading. His color vision was essentially normal (two errors were made on Ishihara plates and testing with Lanthony tritan plates was normal). Goldmann perimetry showed normal outer boundaries. Ophthalmoscopy showed small round heavily pigmented foveal scars surrounded by small drusen-like changes (Figure 2C,D). The appearance of the same macular lesions in multiple family members with an autosomal dominant inheritance pattern led to the diagnosis of NCMD.

**Figure 3:**
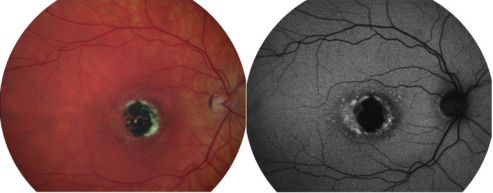
Color (left) and autofluorescence (right) fundus photographs from the right eye of patient II-6, recorded at the age of 35 years. Fluorescence was absent in a hyperpigmented central zone of outer retinal atrophy, whereas an irregular gray to white surrounding zone showed confluent hyperautofluorescence. The general level of fundus autofluorescence outside the macula appeared to be normal.

**Figure 4:**
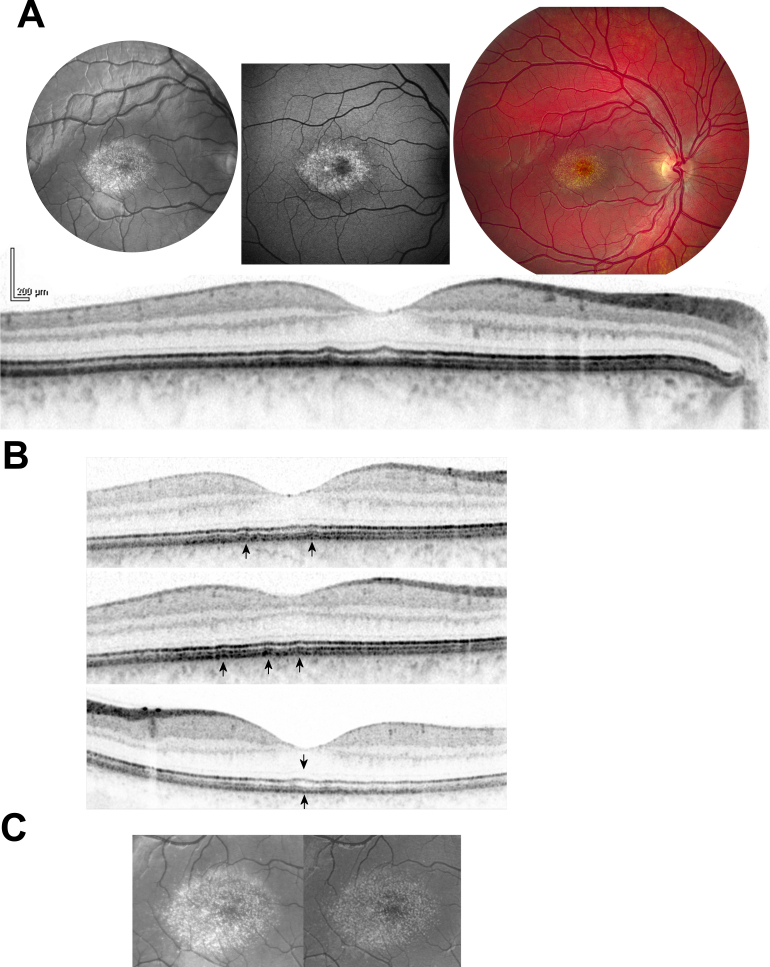
Fundus, autofluorescence, and optical coherence tomography (OCT) images of patients with North Carolina Macular Dystrophy (NCMD). **A**: Fundus images from the right eye of patient III-3, recorded at the age of 19 years. Red-free fundus photographs (upper left) show the dense pattern of approximately 1,500 small semiconfluent yellowish drusen-like elements of 12–25 µm in diameter distributed relatively evenly within one disk diameter of the foveal center, except for a relative sparing of the most central part of the fovea. Autofluorescence fundus photography (upper middle) shows marked hyperfluorescence of the bright drusen-like elements. Some of the smaller elements are likely below the resolution of the autofluorescence imaging system. In and around the foveal center, small, irregular dark patches are seen, which may be attributable to atrophy or to areas of normal fundus that are circumscribed by diffusely hyperfluorescent areas. The diffuse fundus autofluorescence outside the macula does not appear to be elevated compared to healthy subjects of the patient’s age. Color fundus photography (upper right) demonstrates the yellow color of the drusen-like elements being enhanced centrally, presumably by the xanthophyll in Henle’s layer. Horizontal transfoveal optical coherence tomography (OCT, below) shows a normal anatomy of the inner retina, including a normal foveal depression. The photoreceptor-retinal pigment epithelium (RPE) complex appears normal near the optic disk, where all three hyperreflective (dark) and two intervening hyporeflective bands are visible. In the fovea, this pattern is interspersed by localized thickening that involves both the inner and the outer layers of the complex. There are no definite signs of serous detachment of the RPE from Bruch’s membrane, which gives the impression that the basal layers of the photoreceptor outer segment/RPE complex are thickened. Right under the center of the foveola, the inner layers of this complex appear to be locally thickened. **B**: Selected OCTs from patient III-3, recorded at the age of 19 years. Transfoveal (top and bottom) and parafoveal (middle) OCTs from the right eye (top and middle) and the left eye (bottom). The foci of localized thickening of the photoreceptor outer segment/RPE complex (up arrows) were sufficiently hyperreflective to indicate that the outer layers of the complex were thickened or that very small drusen had formed between the RPE and Bruch’s membrane. Serous detachment of the RPE from Bruch’s membrane was not seen. Localized thickening of the inner layers of the complex was seen under the center of the fovea (down arrow). **C**: Stereoscopic fundus photographs from patient III-3, recorded at the age of 19 years. The stereoscopic effect is seen by converging on a point half way between the observer and the figure, so that the two images fuse to a third image in the middle with stereoscopic depth.

The proband (III-3) and her paternal uncle (II-6) were also examined with fundus autofluorescence imaging using confocal scanning laser ophthalmoscopy and optical coherence tomography (OCT, Figure 3 and Figure 4). The bright NCMD elements showed higher autofluorescence than the background (Figure 4A). Semiconfluent dark areas of lower autofluorescence were seen only in the central region of the fovea (Figure 4A). The central hyperpigmented lesions in the uncle were nonfluorescent, whereas the surrounding rim of bright amorphous subretinal material was hyperfluorescent. Infrared images recorded at 790 nm (not shown) showed the same element pattern as in red-free and 488 nm illumination, showing that element hyperreflectivity extends toward the range of the OCT light source, which is centered at 870 nm. On OCT, no definite hyperreflectivity could be seen in the neurosensory retina (Figure 4A), whereas irregularly distributed thickening of the photoreceptor outer segment/retinal pigment epithelium (RPE) complex was seen on multiple sections (Figure 4A,B). There were no signs of accumulation of abnormal material between the base of the RPE and Bruch’s membrane, where the drusen of age-related macular degeneration are found. Stereoscopic montages of red-free fundus images showed the bright elements posterior to and at some distance from the retinal blood vessels (Figure 4C).

### Genetic studies

DNA samples from affected family members were studied with linkage analysis using a stepwise approach. Linkage to the previously reported chromosome 6q locus (MCDR1) was ruled out using a panel of STRP markers (data not shown). Next, a genome-wide scan for linkage was conducted using microarrays of SNP genetic markers on 9 of the 10 affected family members. Analysis of the SNP genotypes identified a region of chromosome 5p with a maximum nonparametric linkage score of 9.88 (p=0.0001). The affected family members shared an allele of 1081 consecutive SNPs that span 13.96 Mbp between the telomere and rs13154455. The linkage to 5p was confirmed by genotyping all 10 affected members of the pedigree with seven STRP markers that span the region (Figure 1). The maximum two-point parametric LOD score of 2.69 (theta=0) was obtained with markers D5S406, D5S1987, and D5S2505.

The linked region for our pedigree partially overlaps with the previously described NCMD locus, MCDR3 [19], and combining these data produces a critical region that is narrowed from 20.0 Mbp to 12.75 Mbp. This critical region contains 55 known candidate genes, of which 30 are expressed in human retina and/or RPE (Table 2), as determined by studies with expression microarrays (data not shown).

**Table 2:** Genes within the linked region on chromosome 5 that are expressed in human retina and/or human RPE.

**Human retina**	**Human RPE**	**Human Retina and RPE**			
ANKRD33B	FAM105A	ADCY2	CTNND2	LPCAT1	NSUN2
MARCH11	FAM105B	ANKH	DAP	MARCH6	PAPD7
	FAM173B	CCT5	FAM134B	MED10	SEMA5A
	IRX2	CLPTM1L	FASTKD3	MRPL36	TRI0
	MTRR	CMBL	FBXL7	MYO10	UBE2QL1
			KIAA0947	NDUFS6	ZNF622

Expression was determined using Affymetrix U133 microarray platform using MAS 5.0 algorithm (data not shown).

## Discussion

The NCMD phenotype is characterized by diverse macular lesions: tiny, drusen-like material in the macular region, foveal pigment epithelial atrophy, or bilateral coloboma-like scars with hyperpigmentations. The lesions are either congenital or appear in early infancy, and the visual acuity in the best eye is often astonishingly good compared with the extent of the macular affection. Among our patients, only one (Figure 1, II-6; Figure 2C,D) had grade 3 lesions, while the remaining nine appeared to have grade 1 lesions. None of the affected family members had any visual complaints and their macular lesions were incidentally discovered with the exception of two family members, who failed routine school vision testing. The appearance of characteristic macular lesions coupled with the autosomal dominant transmission, normal color vision, and normal ffERG, strongly support a diagnosis of NCMD.

While in most pedigrees NCMD is linked to the chromosome 6q locus MCDR1 [5], the macular disease in one NCMD family has been mapped to a chromosome 5p locus, MCDR3 [19]. Here we report a second family with NCMD that is also linked to the MCDR3 locus on chromosome 5p. In addition to confirming the presence of the MCDR3 locus, linkage data from our pedigree allows the MCDR3 critical region to be reduced to a 12.75 Mbp interval that contains 55 genes, 25 of which are expressed in the retina.

Minor phenotypic differences between NCMD that maps to MCDR1 and that which maps to MCDR3 have been reported. Color vision defects were detected in members of the original MCDR3 pedigree, while pedigrees linked to MCDR1 have normal color vision. Progression of disease has been reported in a single member of the original MCDR3 pedigree, but its rare occurrence does not seem to differentiate between pedigrees linked to the MCDR1 and MCDR3 loci [5,19]. Overall, these differences are of minor significance and might represent familial characteristics within a single phenotype. No definite color vision defects and no evidence of disease progression were detected in our pedigree, which is linked to the MCDR3 locus.

The pathogenesis of NCMD is poorly understood. Histopathological studies have provided some insight. Light microscopy of the eyes from a 72-year-old woman with grade 2 NCMD showed complete loss of photoreceptor cells and the underlying RPE, Bruch’s membrane attenuation, and marked atrophy of the choriocapillaris [29,30]. The same anatomic lesions have been demonstrated by in-vivo OCT techniques [13,31]. Fundus hyperautofluorescence, as seen in the drusen-like material in NCMD in patient III-3, is not necessarily evidence of the presence of any specific biochemical entity, but in several other retinal diseases it is attributed to the fluorophore A2E, a retinoid dimer that accumulates in conditions where the visual cycle is impaired. Notable conditions where fundus hyperfluorescence is higher than normal include Stargardt’s disease [32] and age-related macular degeneration [33]. In these two conditions, hyperautofluorescence is caused by increased fluorescent content in the RPE. In retinitis pigmentosa, as in age-related macular degeneration, higher than normal fundus autofluorescence can be seen on the border between areas of degenerated and preserved photoreceptors [34]. While increased fluorophore content is one possible explanation for this phenomenon, the optical unmasking of a normal fluorophore content by loss of natural retinal pigments such as melanin is also a theoretical possibility.

Structural elements of the retina that appear bright in infrared fundus photographs should show up on infrared OCT, but in the case of NCMD elements, the lateral resolution of the instrument may be insufficient to resolve the individual elements. The lack of abnormal hyperreflectivity in the neurosensory retina in the young patient we investigated by spectral-domain OCT suggests that the bright elements could be located in the RPE and that their presence could be masked by the high reflectivity of the normal RPE. The bumpy, irregular contour of the RPE noted on several of the OCT scans also suggests that the photoreceptor outer segment/RPE- complex is the primary target of disease in NCMD. This would be compatible with the eventual development of geographic outer retinal atrophy in the later stages of the disease.

Decaying tissue of the outer retina is often seen outside the rim of a chorioretinal scar such as the central hyperpigmented scar in patient II-6. Such bright, hyperautofluorescent, amorphous material may be a nonspecific product of cell death.

In this report, we present a second NCMD family linked to chromosome 5p that confirms the previous discovery of the MCDR3 locus. Combining the linkage studies of our family and the original MCDR3 produced a new critical region of 12.75 Mbp that contains a gene that causes NCMD. These results represent important steps toward the discovery of genes that are capable of causing NCMD, which will provide novel insights into the pathogenesis of this condition.
